# Early radiation effects on immediate breast reconstruction: A comparative analysis of 15- and 5-fraction postmastectomy radiotherapy regimens

**DOI:** 10.1016/j.jpra.2026.02.011

**Published:** 2026-02-16

**Authors:** Sarah Omar, Abdou M.A. Darwish, Krzysztof Sosnowski, Simon Russell, Ayman Noaman El-Henawy, Ashraf Othman, Charles Malata

**Affiliations:** a Department of Plastic Surgery, Minia University Hospital, Kornish El Nile Street, Minia, Egypt; b School of Clinical Medicine, University of Cambridge, Hills Road, Cambridge, United Kingdom; c Department of Oncology, Addenbrooke’s Hospital, Hills Road, Cambridge, United Kingdom; d Department of Plastic Surgery, Kasr El Aini University Hospital, Cairo University, El-Quasr-Al-Aini Street, Cairo, Egypt; e Department of Clinical Pathology, Minia University Hospital, Kornish El Nile Street, Minia, Egypt; f Cambridge Breast Unit, Addenbrooke’s Hospital, Hills Road, Cambridge, United Kingdom; g Anglia Ruskin University School of Medicine, Bishop Hall Lane, Cambridge & Chelmsford, United Kingdom; h Department of Plastic Surgery, Addenbrooke’s Hospital, Hills Road, Cambridge, United Kingdom

**Keywords:** Breast reconstruction, Postmastectomy radiotherapy, Ultra-hypofractionation, Hypofractionation, Acute toxicity, RTOG/EORTC

## Abstract

**Background:**

Modern postmastectomy radiotherapy (PMRT) increasingly employs ultra-hypofractionated regimens (26 Gy/5 fractions) as an alternative to standard hypofractionation (15 fractions/40–42 Gy). However, evidence on their safety in the reconstructive setting remains limited. This study compares acute toxicity outcomes between these regimens and a non-irradiated control group following immediate breast reconstruction.

**Methods:**

A single-center cohort study included 203 immediate breast reconstructions in 168 patients (implant = 59, DIEP = 47, LD = 33, BCS = 64) performed between July 2024 and July 2025. Patients received either 3-week (*n* = 63) or 5-day (*n* = 50) PMRT, or no radiotherapy (*n* = 90). Acute toxicity (≤3 months) was graded using the RTOG/EORTC scale. Kruskal-Wallis and ordinal logistic regression models were used to assess the influence of radiotherapy exposure, dose, and reconstruction type on toxicity severity and intervention requirements.

**Results:**

Across the cohort, most reconstructions exhibited mild toxicity (47.8%), with more severe reactions (Grade 3–4) limited to 7.4% of cases. Radiotherapy exposure significantly increased toxicity severity (*H* = 30.8, *p* < .001), whereas differences between 3-week and 5-day regimens were non-significant (*p* > .05). Regression models confirmed RT exposure, particularly conventional 3-week fractionation, was the main predictor of higher RTOG/EORTC scores (*p* < .001), while reconstruction type alone had no significant independent effect. The 5-day regimen had a comparable acute toxicity profile to the 3-week regimen.

Erythema and oedema were the most frequent manifestations, both predominantly low-grade. Intervention rates were low across groups (<7%), and no increase in acute surgical management was observed with ultra-hypofractionated RT.

**Conclusions:**

Both 5-day and 3-week PMRT regimens were associated with mild, self-limiting acute toxicity, with no evidence of higher complication or intervention rates using ultra-hypofractionation. These findings support the 5-fraction schedule as a safe, efficient alternative to standard PMRT in appropriately selected reconstruction patients with a comparable, if not milder, acute toxicity profile. Long-term follow-up is warranted to evaluate chronic effects and reconstructive durability.

## Introduction

Radiotherapy (RT) remains a cornerstone of breast cancer management, substantially reducing locoregional recurrence and breast cancer-related mortality.^1^ It is most commonly administered as adjuvant therapy following surgical intervention, either as whole-breast irradiation (WBI) following breast-conserving surgery (BCS) or post-mastectomy radiotherapy (PMRT).^1^^,^^2^ The latter is indicated in patients at higher risk of locoregional recurrence, such as those with positive axillary lymph nodes or involved surgical margins. Depending on disease extent and nodal status, radiation fields may encompass the chest wall, axillary, supraclavicular, and internal mammary nodal regions.^2^^,^^3^

Despite being a well-tolerated treatment modality, RT is associated with both acute and late toxicities. Acute toxicity manifests within 3 months of treatment completion, whereas late effects occur months to years later.^4^ When applied to reconstructed breasts, RT introduces additional challenges due to the altered vascularity and tissue composition of reconstructed sites.^5^^,^^6^ Radiation effects on the mastectomy skin flap, implant capsule, or autologous tissue closely resemble those observed in native breast tissue but can be amplified by surgical scarring, reduced perfusion, or the presence of prosthetic materials.

Moderately hypofractionated regimens (≈40 Gy/15 fractions) have demonstrated reconstructive complication rates comparable to conventional 25-fraction schedules.^7^^,^^8^ However, the reconstructive safety profile of the 5-fraction ultra-hypofractionated regimen remains poorly defined. While the oncologic equivalence of ultra-hypofractionated PMRT has been firmly established, data regarding its acute toxicity, particularly in reconstructed breasts, remain limited.^9^, ^10^^–^^11^

This study, therefore compared two fractionation regimes with respect to the rate and nature of acute radiation-related complications in immediate breast reconstruction. The incidence, timing, and severity of acute (≤3 months) toxicity were evaluated using the RTOG/EORTC grading system, with comparisons made by RT regimen and reconstruction type.^12^ Intervention types and frequencies for managing acute complications were recorded to assess variation between RT regimens.

## Methods

This was a retrospective, non-randomized, longitudinal comparative cohort study with prospective analysis of extracted data. The study compared radiation-induced acute toxicity among three cohorts differing in PMRT exposure and dose/fractionation.

Data were obtained from Addenbrooke’s Hospital (Cambridge University Hospitals NHS Foundation Trust), using customized workbenches in the Epic electronic medical record system, which was created in Madison, Wisconsin, USA.^13^ These reports identified patients who underwent mastectomy and immediate breast reconstruction between November 2014 and July 2023, allowing 2 years of follow-up. All data extraction, validation, and photographic analysis were conducted prospectively once the cohort was identified.

**Figure 1:**
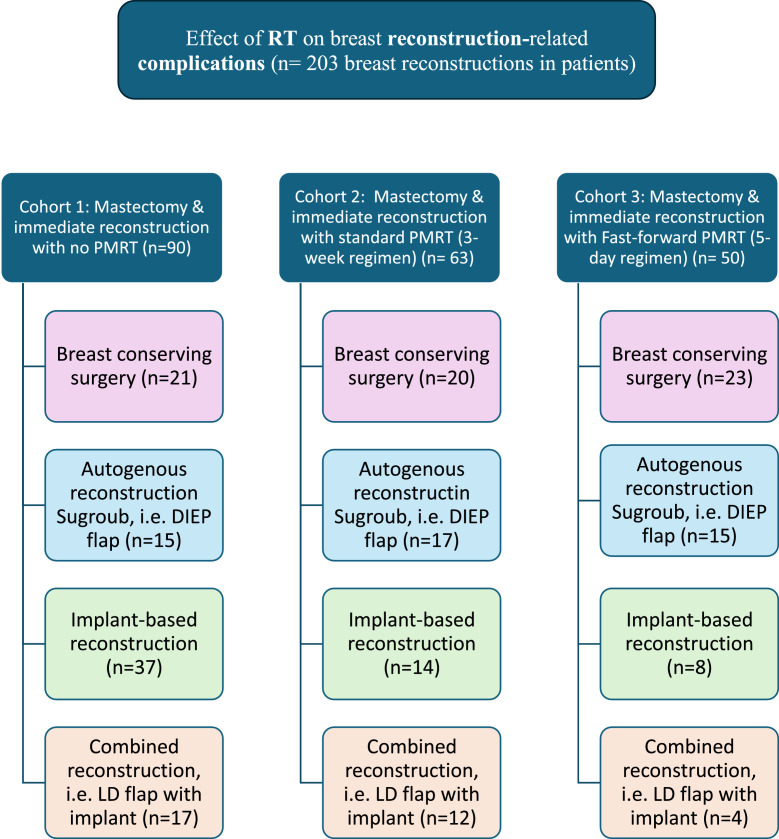
Graphical representation of the three main cohorts classified by radiotherapy exposure, along with their respective subgroups based on type of reconstruction.

Participants were allocated into three main cohorts based on PMRT exposure and fractionation schedule (Figure 1):

• Group 1 – No RT (Control): No adjuvant radiotherapy after mastectomy and immediate reconstruction.
• Group 2 – Standard Hypofractionated RT: 40–42 Gy delivered in 15 fractions over 3 weeks.^11^
• Group 3 – Ultra-hypofractionated RT (FAST-Forward regimen): 26 Gy delivered in 5 fractions over 1 week.^10^^,^^11^

Allocation was non-randomized and based on historical oncologic decisions. To minimize sampling bias during data extraction, random selection (via random number generation) was used in subgroups with large eligible populations, whereas all eligible patients were included where case numbers were limited.

Each main cohort comprised four reconstruction-type subgroups:

1. Breast-Conserving Surgery (BCS) with local flap or reduction techniques,
2. Implant-Based Breast Reconstruction (IBBR),
3. Autologous Breast Reconstruction (ABR) such as DIEP or SIEA flaps, and
4. Combined Reconstruction using LD or TDAP flaps with implants.

All breast cancer patients who underwent mastectomy and immediate reconstruction between November 2014 and July 2023 were included, expect those with previous breast or chest wall radiotherapy to the same side of interest.

### Data collection and timeframes

Relevant clinical data and standardized photographs were collected for the acute evaluation window (≤3 months).

• For irradiated patients (Groups 2 and 3), the acute window encompassed the period during radiotherapy and the subsequent 3 months after completion^4^^,^^12^
• For the non-irradiated control cohort (Group 1), the postoperative period during the first 3 months following reconstruction was examined.

Each reconstruction received an RTOG-EORTC score representing the highest recorded grade of acute toxicity within the observation period. When explicit toxicity grades were documented by the treating radiation oncologist, these were directly adopted. When not explicitly graded, detailed clinical descriptions from follow-up records were reviewed and scored by the first author according to RTOG-EORTC criteria. All acute radiation-related manifestations were systematically identified and tabulated to enable subsequent frequency and correlation analyses.

The number and type of interventions required to manage acute radiation-related complications, were documented. These data were reserved for inferential analysis and discussion of management strategies.

## Statistical analysis

All statistical analyses were performed using JASP (version 0.95.3), which was developed at the University of Amsterdam.^14^

Descriptive statistics were used to summarize baseline characteristics and complication frequencies, whilst the Kruskal-Wallis tests compared ordinal or non-parametric continuous data between groups. Ordinal and nominal regression analyses were used to assess associations between RT regimen, reconstruction type, and toxicity grade. Statistical significance was set at *p* < .05.

## Results

### Study cohorts and descriptive characteristics

**Figure 2:**
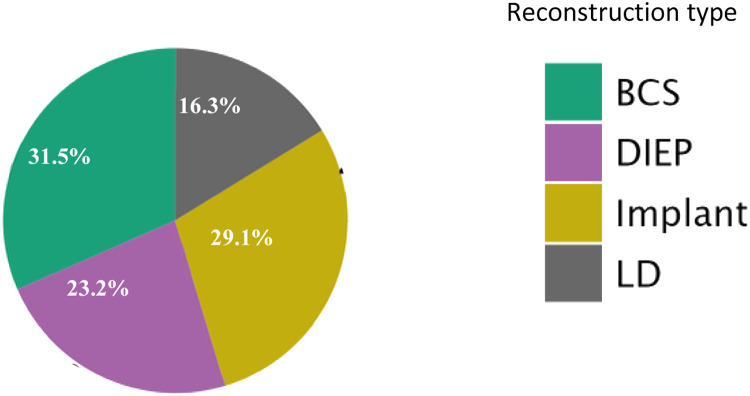
A pie chart representation of the different reconstruction types in the study samples in percentages. The smaller size of the LD flap group, relative to the other reconstruction groups, reflects the LD flap fall out of favor, in breast reconstruction, in recent years.

This study comprised 203 immediate breast reconstructions in 168 women (35 bilateral; mean age = 52.5 years, range 23–83) performed at the time of mastectomy or lumpectomy. The reconstructions (*n* = 203), in 29.1% (*n* = 59) were implant-based, 47 (23.1%) used DIEP free flaps, 33 (16.2%) used LD flaps, whilst 64 (31.5%) followed BCS with local reconstruction (Figure 2).

**Figure 3:**
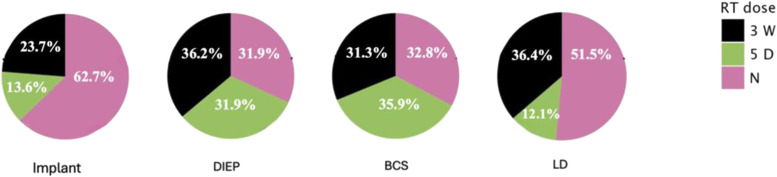
A pie chart representation of distribution of exposure to RT, and RT dose (if applicable) in the study sample in percentages, in every reconstruction category. The smaller number of patients in the implant and LD groups who received the ultrahypofractionated (5-day) radiotherapy regimen—compared to the more balanced distribution between 3-week and 5-day RT in the DIEP and BCS groups—may reflect a clinical tendency to avoid this newer fractionation approach in implant-based reconstructions. This observation warrants inferential analysis to explore any potential association.

Post-mastectomy radiotherapy (RT) was delivered in 113 breasts (55.6%), subdivided as 63 (31.0%) 3-week RT, 50 (24.6%) 5-day RT, and 90 (44.3%) no RT (Figure 3). Analyses were conducted per reconstruction to account for bilateral cases, acknowledging intra-patient non-independence as a confounder. Non-parametric and generalized regression models were applied to reduce within-subject correlation bias.

### Reconstruction and oncologic procedure characteristics

**Figure 4:**
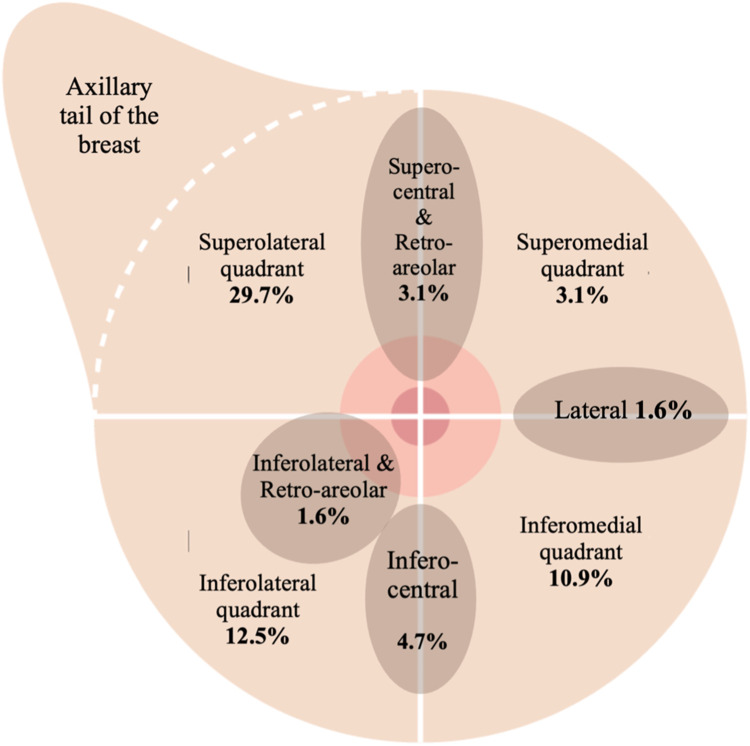
A diagram illustrating distribution of tumor excision sites in the breast conserving surgery group. The superolateral quadrant had the highest predominance of tumors.

Skin-sparing mastectomy predominated in implant (88.1%), DIEP (83.0%), and LD (93.9%) reconstructions. Within BCS cases, 32.8% were symmetrizing reductions, most often in the superolateral quadrant (29.7%) (Figure 4). Nearly all LD flaps (96.9%) were pedicled and combined with implants (93.9%), reflecting their hybrid use, while DIEP reconstructions included 10 bipedicled flaps (21.3%), illustrating procedural diversity.

### Assessment of acute toxicity

**Figure 5:**
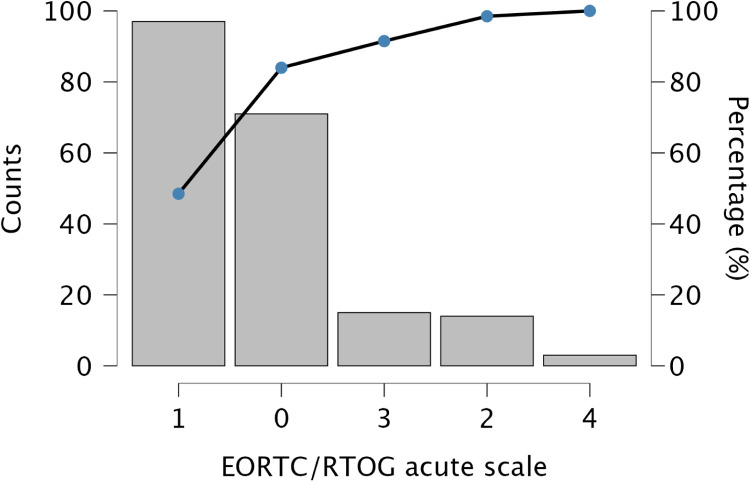
Pareto chart illustrating the distribution of EORTC/RTOG scores for acute radiotherapy-related manifestations in the overall study population.

Across the cohort (*n* = 203), 35.5% showed no toxicity, 47.8% mild, 6.9% moderate, 7.4% severe, and 1.5% very severe (Figure 5). Mild reactions predominated: 82% in the 5-day and 72.1% in the 3-week groups. No 5-day cases were toxicity-free, indicating universal but mild effects. Grade 3-4 events occurred only in the 3-week group (11.4%). In non-irradiated cases, 22.5% displayed mild postoperative inflammation rather than radiation effects. By reconstruction type, 3-week RT showed broader score variation, especially in DIEP and LD flaps, while 5-day RT produced a consistent low-grade profile.

### Effect of radiotherapy exposure and dose

Both Kruskal-Wallis and ordinal logistic regression analyses confirmed a strong RT-toxicity relationship. The Kruskal-Wallis test was highly significant (*H* = 30.80, *p* < .001, *ε*² = 0.444), showing higher scores in both RT regimens versus no RT (*p* < .001) but no difference between regimens (*p* > .05). Ordinal regression (*χ*² = 110.4, *p* < .001) supported these findings, showing no significant difference between 5-day and 3-week regimens (Estimate = 0.134, *p* = .734), while no RT had significantly lower odds of toxicity (Estimate = 3.647, *p* < .001). A multinomial model (overall *p* < .001) suggested slightly higher toxicity with 3-week RT (*p* = .026) but no significant 5-day vs. no-RT difference (*p* > .05). Overall, RT, particularly conventional 3-week PMRT, was the key determinant of acute toxicity, with ultra-hypofractionation showing comparable or milder effects.

### Effect of reconstruction type

An ordinal regression indicated significance (*p* = .003), though unstable coefficients limited interpretation. A confirmatory Kruskal–Wallis test showed no between-type differences (*p* > .05). Thus, reconstruction type alone did not meaningfully affect acute toxicity severity.

### Combined effect of radiotherapy and reconstruction type

**Figure 6:**
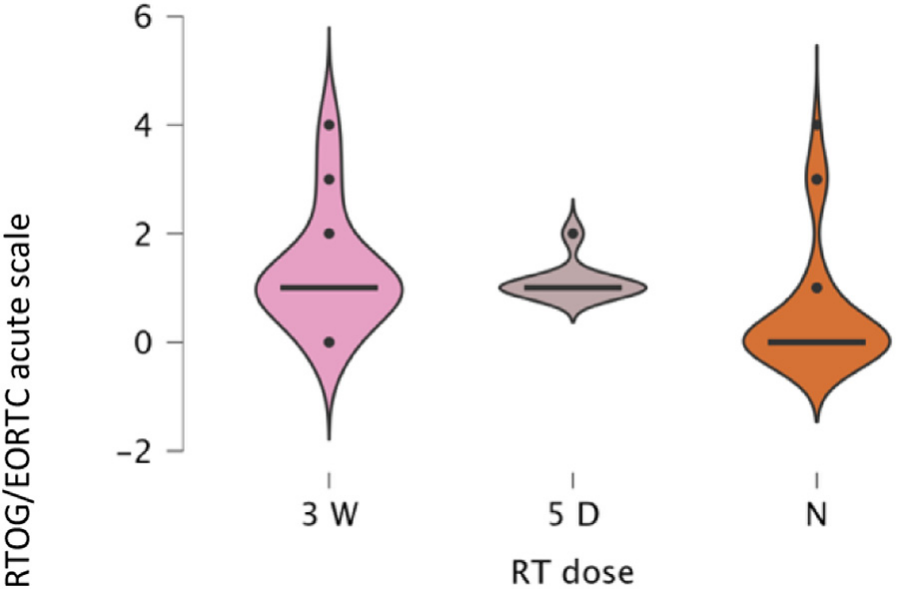
A violin boxplot representing the distribution of RTOG/EORTC scores across the three cohorts (no RT, 5-day RT, and 3-week RT) in implant cases.

**Figure 7:**
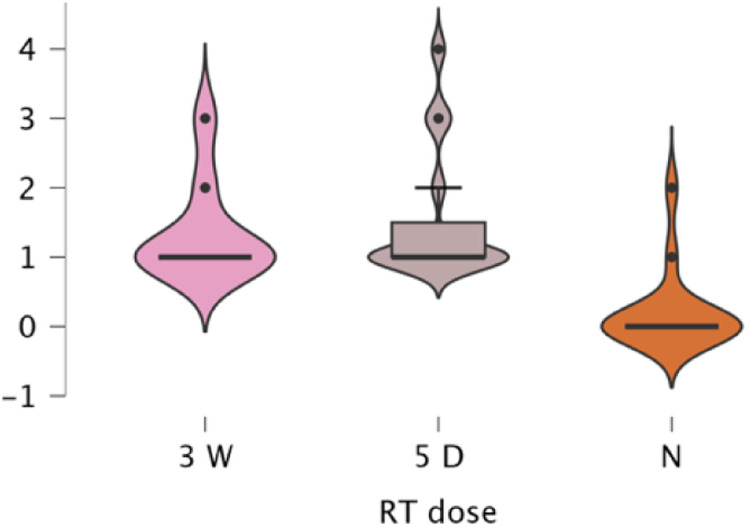
A violin boxplot representing the distribution of RTOG/EORTC scores across the three cohorts (no RT, 5-day RT, and 3-week RT) in DIEP cases.

**Figure 8:**
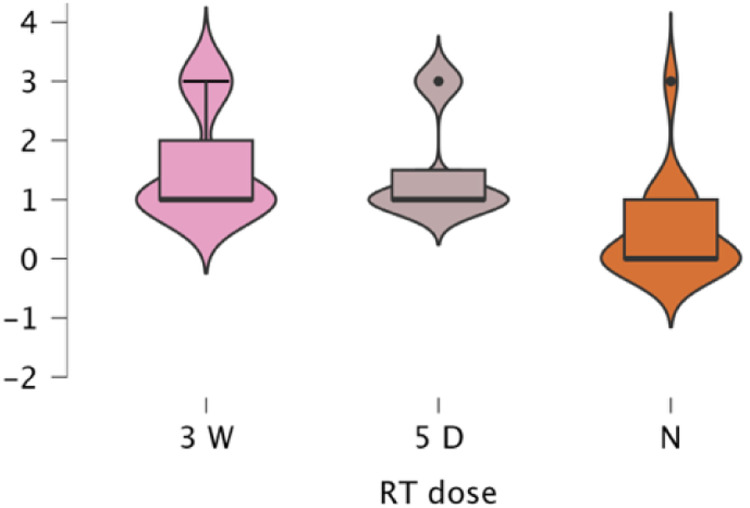
A violin boxplot representing the distribution of RTOG/EORTC scores across the three cohorts (no RT, 5-day RT, and 3-week RT) in LD cases.

**Figure 9:**
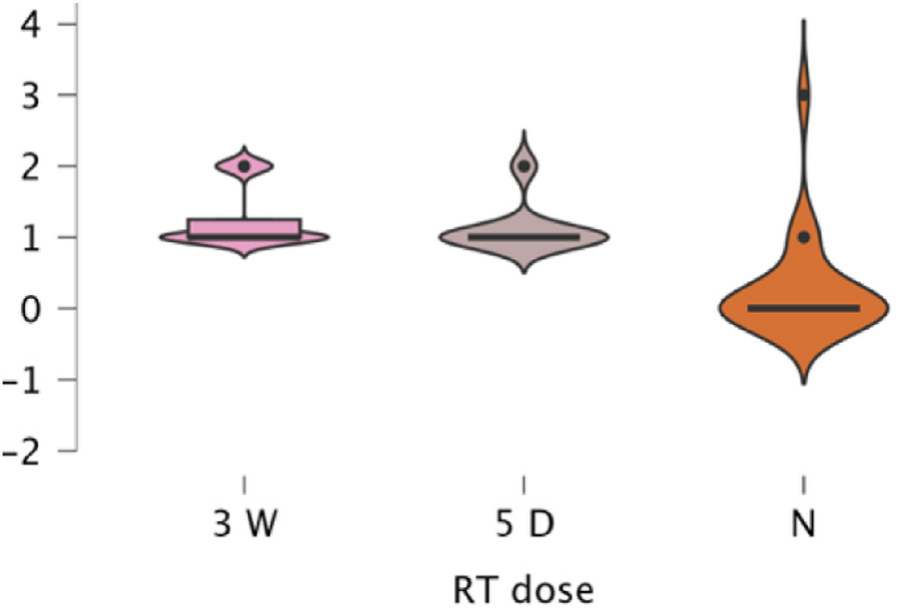
A violin boxplot representing the distribution of RTOG/EORTC scores across the three cohorts (no RT, 5-day RT, and 3-week RT) in BCS cases.

Subgroup analyses confirmed significant RT effects within all reconstruction categories, implant (*p* < .001, *ε*² = 0.280), DIEP (*p* < .001, *ε*² = 0.562), LD (*p* = .001, *ε*² = 0.449), and BCS (p < .001, *ε*² = 0.539). Across all types, toxicity was lowest for no RT (mean 0.20–0.54) and higher for both RT regimens (1.13–1.54). Differences between 3-week and 5-day RT were small and inconsistent (e.g., Implant 1.31 vs. 1.13; DIEP 1.53 vs. 1.35). Post hoc analyses confirmed higher toxicity for both RT regimens versus no RT (*p* = .002–.001). Ordinal regression (*χ*² = 114.2, *p* < .001) identified RT exposure as the dominant predictor (3-week Estimate = 3.94, *p* < .001; 5-day Estimate = 0.35, *p* = .583) with no significant RT × reconstruction interactions. A multinomial model (*χ*² = 197.8, *p* < .001) reaffirmed these results, conventional RT drove the highest toxicity, while the 5-day regimen remained statistically comparable and generally milder (Figure 6, Figure 7, Figure 8, Figure 9).

### Acute toxicity manifestations

#### Erythema

**Figure 10:**
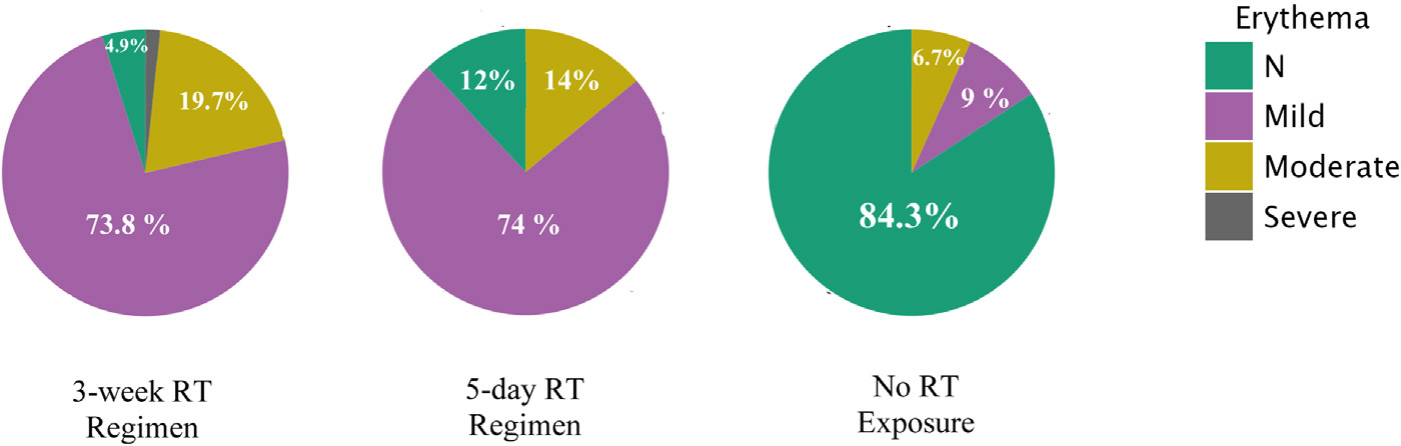
Pie chart illustrating the distribution of erythema severity grades (none, mild, moderate, severe) across the three treatment cohorts, after exclusion of reconstructions that lacked sufficient relative data on their files. The 5-day cohort shows a favorable erythema profile, closely mirroring the 3-week group in terms of mild/moderate distribution but with no severe cases, and more cases experiencing no reaction, highlighting its relative safety. Meanwhile, erythema in the no-RT group, including moderate cases, suggests other contributors to skin irritation post-treatment.

Erythema was the most frequent manifestation (Figure 10). In the 3-week cohort (*n* = 61), mild erythema occurred in 73.8%, moderate in 19.7%, severe in 1.6%, and none in 4.9%. In the 5-day group (*n* = 50), mild erythema affected 74%, moderate 14%, none 12%, and none were severe. Among non-irradiated reconstructions (*n* = 89), erythema was absent in 84.3%, mild in 9%, and moderate in 6.7%. The Kruskal–Wallis test confirmed a strong dose effect (*χ*²(2) = 30.80, *p* < .001, *ε*² = 0.478); both RT regimens had higher erythema than no RT (*p* < .001), with no difference between regimens (*p* > .300). Mean erythema: 3-week = 1.18, 5-day = 1.02, no RT = 0.23. Reconstruction type showed a smaller effect (*χ*²(3) = 9.36, *p* = .025), with BCS higher than implant (pBonf = .012). Within subgroups, RT dose remained a strong predictor (all *p* < .001). Ordinal regression (*χ*²(586) = 120.5, *p* < .001) confirmed RT dose as dominant; 5-day ≈ 3-week, both > no RT. Reconstruction type and interactions were non-significant. A multinomial model (*χ*²(564) = 166.7, *p* < .001) yielded identical conclusions.

#### Oedema and other acute manifestations

Oedema was the second most frequent finding. In the 3-week group, mild oedema affected 77%, moderate 4.9%; in the 5-day group, 46% mild, 4% moderate; and in no RT, only 10% mild or moderate. Desquamation/blistering occurred in 8.1% (3-week), 10.0% (5-day), 4.4% (no RT). Infection/cellulitis remained rare (3.2%, 2.0%, 4.4%), and pain modest (6.4%, 8.0%, 2.2%). Other manifestations, ulceration, necrosis, hyperpigmentation, or contracture, were isolated and generally mild. Kruskal–Wallis testing showed a strong RT dose effect on oedema (*H* = 70.61, *p* < .001, *η*² = 0.357), with a gradient of 3-week > 5-day > no RT. Reconstruction type also influenced oedema modestly (*H*(3) = 9.86, *p* = .020), with DIEP flaps showing higher scores (*p* = .013). Subgroup analyses confirmed consistent RT-driven trends across all types (*p* < .001). Ordinal regression (*χ*²(385) = 97.19, *p* < .001) again identified RT dose as the key predictor, both 5-day (Estimate = 2.36, *p* < .001) and 3-week (Estimate = 4.84, *p* < .001) exceeding no RT. Reconstruction effects and limited interactions (e.g., DIEP × 5-day RT, *p* = .015) were secondary. Chi-square tests showed no significant associations between RT exposure and other acute complications (all *p* > .05), with only implant exposure/dehiscence trending toward significance (*p* = .078). Most non-significant results suggested low clinical impact across minor complications.

### Interventions for acute complications

**Figure 11:**
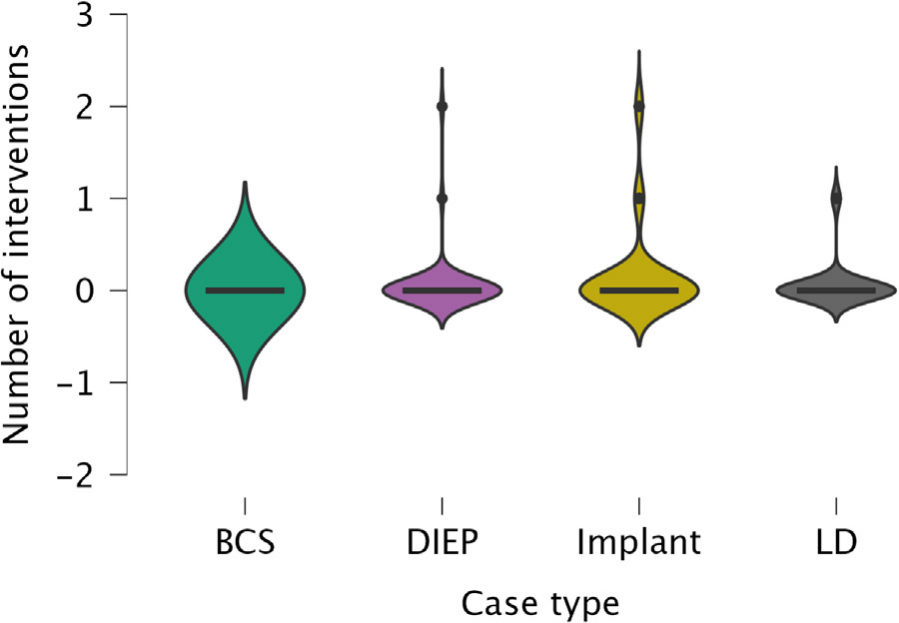
A violin boxplot representing the distribution of frequencies of “interventions needed for acute complications” among different reconstructive groups.

Interventions were rare across all cohorts (Figure 11). In the 3-week group (*n* = 63), 61 patients (96.8%) developed toxicity, yet only 3 reconstructions (4.9%) required intervention: one hospital admission for cellulitis, one capsulectomy for early contracture, and one implant explantation for exposure. In the 5-day group (*n* = 50), all had mild toxicity, but only 2 (4%) required procedures: one LD flap salvage and one infection-related washout. Among non-irradiated cases (*n* = 90), 20 (22.2%) showed mild complications; 6 (6.6%) needed interventions, three single-stage (implant exchange, skin graft, IV antibiotics) and three staged (two implant explantations culminating in DIEP salvage, one explantation with subsequent implant replacement). Overall, <7% of reconstructions required intervention, with no clear RT dose effect. Kruskal–Wallis testing confirmed no link between RT dose and intervention number (*χ*²(2) = 0.573, *p* = .751, *ε*² = 0.003). Reconstruction type, however, significantly influenced intervention rates (*χ*²(3) = 8.663, *p* = .034, *ε*² = 0.044), with implants needing more than BCS (*p* = .021). An ordinal regression (*χ*² = 23.05, *p* = .017) showed reconstruction type and RT dose jointly predicted intervention frequency, driven mainly by the combination of implant-based no-RT cases, which required fewer interventions than all other subgroups.

## Discussion

Across 203 immediate reconstructions, radiotherapy exposure was the main predictor of acute skin and soft-tissue toxicity, whereas reconstruction type had a lesser effect. Both regimens increased erythema and oedema relative to controls, but reactions were generally mild, self-limiting, and clinically manageable. No significant difference was observed between the two RT schedules, suggesting that the 5-fraction regimen is at least as safe, and possibly slightly less toxic, than the 15-fraction protocol.

Findings align with growing evidence that modern hypo- and ultra-hypofractionated breast RT achieve comparable early toxicity to conventional fractionation. The predominance of mild erythema and oedema, and the rarity of severe (≥Grade 3) reactions, mirror results from the FAST-Forward trial and recent meta-analyses, all showing equivalent or reduced acute toxicity with 5-fraction regimens.^9^^,^^15^^,^^16^

The strong relationship between RT exposure and acute toxicity reflects the radiosensitivity of mastectomy skin flaps and reconstructed tissues. Yet, the absence of difference between the two modern RT regimens suggests that fractionation schedule—rather than total dose, is not a major determinant of early reactions within current therapeutic ranges. This supports Royal college of Radiologists (RCR), (2024) guidance recognizing both 15- and 5-fraction schedules as safe standards. The consistently mild presentations among irradiated cohorts also highlight advances in image guidance, bolus control, and dose homogeneity.

The results concur with Park et al.^16^, who found no increase in acute complications with hypofractionated (15 fractions over 3 weeks) versus conventional PMRT (25 fractions over 5 weeks) after implant reconstruction, and with Hayashi et al.,^15^ who reported similar early complication rates between 15- and 5-fraction regimens. By applying RTOG/EORTC grading to a mixed reconstructive cohort (implant, DIEP, LD, and BCS), this study extends prior findings and confirms that acute toxicity remains mild across techniques.

Although autologous reconstructions showed slightly higher erythema and oedema scores than implant-based cases, these differences were minor and clinically negligible. Multivariable modeling confirmed that reconstruction type contributed minimally once RT exposure was accounted for, consistent with, who found reconstruction method did not significantly influence early RT toxicity.^17^ While radiotherapy exposure was the primary determinant of acute toxicity regardless of reconstruction method, the consistency of mild reactions across implant-based, autologous, and hybrid techniques suggests that modern RT planning adequately accommodates diverse tissue compositions. The slightly higher oedema in DIEP flaps and increased intervention rates in implant reconstructions likely reflect inherent tissue characteristics, autologous tissue’s inflammatory response and prosthetic materials’ susceptibility to infection and capsular reactions, rather than fractionation-related differences.

The lack of difference between the 3-week and 5-day regimens parallels FAST-Forward outcomes, showing equivalent tumor control and tissue tolerance.^18^ This confirms that the 5-fraction schedule is safe for reconstructed breasts, provided surgical healing is complete. Of course, these outcomes are dependent on careful planning to optimize flap and implant dose distribution.

### Acute toxicity patterns

Erythema and oedema were the most frequent toxicities, consistent with acute radiodermatitis patterns.^12^^,^^19^^,^^20^ Most cases were mild and transient, reflecting effective dose modulation and modern delivery precision. Desquamation, infection, and pain were infrequent and showed no regimen-related differences, confirming that severe acute reactions are rare under current techniques.

The slightly narrower toxicity range in the 5-day cohort may reflect biological equivalence with reduced cumulative stress on normal tissues, consistent with the radiobiological modeling of the FAST and FAST-Forward trials.^10^^,^^18^

### Interventions and clinical relevance

Interventions were uncommon (<7%) and typically conservative, with most acute toxicities resolving without escalation. The few cases requiring hospitalization or surgery were isolated and not clearly regimen-related. Notably, the highest intervention rate occurred in the non-irradiated group (6.6%), largely from postoperative, non-radiation complications, demonstrating that surgical and patient-specific factors often drive intervention need.

Implant reconstructions required more interventions than BCS, consistent with higher baseline risks of infection and wound tension.⁶ However, ultra-hypofractionation did not amplify these risks, confirming its reconstructive safety.

### Clinical implications

The 5-day ultra-hypofractionated PMRT appears safe in reconstructed breasts, showing no excess acute morbidity compared with the 3-week schedule. When oncologic criteria permit, the 5-fraction regimen offers an efficient alternative, reducing treatment time, patient visits, and healthcare resource use, without compromising short-term safety. This is especially relevant post-pandemic, as systems continue to prioritize efficiency.^21^^,^^22^

For reconstructive surgeons and radiation oncologists, these results support shared decision-making that balances oncologic control with reconstructive integrity. The observed correlation between radiotherapy exposure and acute toxicity underscores the importance of coordination between reconstructive and radiation oncology teams. Optimal timing of reconstruction relative to planned PMRT, selection of appropriate reconstructive techniques for irradiated fields, and shared patient counseling regarding expected acute reactions minimize complications while preserving oncologic and aesthetic outcomes. Patients with well-healed surgical sites and adequate coverage appear suitable for the shorter regimen. Comparable tolerance across reconstruction types broadens eligibility for ultra-hypofractionated PMRT and may streamline multidisciplinary coordination.

### Study limitations and future directions

The retrospective design introduces potential selection bias, as RT regimens were determined clinically before study conception, and bilateral reconstructions may share biological factors affecting outcomes. Unequal subgroup sizes, particularly the smaller LD/5-day cohort, may have reduced power to detect minor effects. The 3-month follow-up window captured acute reactions but excluded late sequelae such as fibrosis, contracture, or aesthetic changes.

Future research should confirm these findings prospectively in larger, multicenter cohorts using patient-reported outcomes and imaging-based assessments. Integration of RT dosimetry data (e.g., dose to skin and reconstructive planes) and long-term evaluation of flap perfusion and reconstructive durability will be essential for establishing comprehensive safety profiles.

## Conclusion

In this cohort of 203 reconstructed breasts, radiotherapy exposure was the main determinant of acute toxicity; however, both 3-week and 5-day PMRT regimens resulted predominantly in mild, self-limiting reactions. The ultra-hypofractionated 5-fraction schedule showed no increase in toxicity or intervention rates compared with the 15-fraction regimen, confirming its safety in reconstructed breasts.

Given its shorter treatment time, lower resource demand, and comparable safety, the 5-fraction regimen is a practical alternative to standard PMRT for appropriately selected reconstruction patients. Long-term follow-up is warranted to evaluate late effects and reconstructive durability.

## Ethical approval and consent

This study was registered and approved as a clinical audit project under Audit Project Number ID7249 by the Audit and Clinical Governance Committee (approval number PRN 13249) at Addenbrooke’s Hospital, Cambridge University Hospitals NHS Foundation Trust. All patients included in the study provided informed consent for their clinical data to be used anonymously for research and audit purposes. Additionally, separate written consent was obtained for the use of any clinical photographs included in the study.

## Funding

No funding was received.

## Data availability statement

Data sharing is not applicable to this article.

## Conflict of interest

No potential competing interest was reported by the authors.
